# Flagellimonas ulvae sp. nov. and Flagellimonas rhodophyticola sp. nov., isolated from marine algae

**DOI:** 10.1099/ijsem.0.006996

**Published:** 2025-12-09

**Authors:** Hui Seong Won, Dong Min Han, Jeong Min Kim, Hülya Bayburt, Byeong Jun Choi, Zhe-Xue Quan, Che Ok Jeon

**Affiliations:** 1 Department of Life Science, Chung-Ang University, Seoul 06974, Republic of Korea; 2 School of Life Sciences, Fudan University, Shanghai 200438, PR China

**Keywords:** *Flagellimonas rhodophyticola*, *Flagellimonas ulvae*, *Flavobacteriaceae*, marine algae, new taxa

## Abstract

Two Gram-stain-negative, strictly aerobic, non-motile, rod-shaped bacteria, designated strains S174ᵀ and W118ᵀ, were isolated from the marine green alga *Ulva prolifera* and the marine red alga *Schizymenia dubyi*, respectively, collected in South Korea. Both strains exhibited catalase- and oxidase-positive activities and grew optimally at 25 °C, pH 8.0 and 2.0% (w/v) NaCl. The major fatty acids (>10%) in both strains were iso-C_15:1_ G, iso-C_15:0_ and iso-C_17:0_ 3-OH, with phosphatidylethanolamine as the major polar lipid and menaquinone-6 as the predominant respiratory quinone. The genomic DNA G+C contents, based on whole-genome sequences, were 39.1 mol% for both strains. Strains S174ᵀ and W118ᵀ shared 93.6% 16S rRNA gene sequence similarity, 72.7% average nucleotide identity (ANI) and 17.5% digital DNA–DNA hybridization (dDDH), supporting their classification as distinct species. Phylogenetic analyses based on 16S rRNA gene and genome sequences placed both strains in distinct lineages within the genus *Flagellimonas*. Strains S174ᵀ and W118ᵀ were most closely related to *Flagellimonas meridianipacifica* SW027ᵀ (97.2%) and *Flagellimonas onchidii* XY-359ᵀ (97.9%), respectively, and ANI and dDDH values with other type strains were below 84.6 and 28.2%, respectively, confirming their novelty. Based on phenotypic, chemotaxonomic and genomic features, strains S174ᵀ and W118ᵀ represent two novel species of the genus *Flagellimonas*, for which the names *Flagellimonas ulvae* sp. nov. (S174ᵀ=KACC 24029ᵀ=JCM 37803ᵀ) and *Flagellimonas rhodophyticola* sp. nov. (W118ᵀ=KACC 24030ᵀ=JCM 37802ᵀ) are proposed.

## Introduction

The genus *Flagellimonas*, belonging to the family *Flavobacteriaceae* within the phylum *Bacteroidota*, was first proposed by Bae *et al.* [1] in 2007 with *Flagellimonas eckloniae* as the type species. In 2024, Novoa *et al.* [2] reclassified 36 species previously assigned to the genera *Allomuricauda* and *Muricauda* into the genus *Flagellimonas*. As of 2 August 2025, the genus comprises 45 validly published and 3 invalidly published species (https://lpsn.dsmz.de/genus/flagellimonas), primarily isolated from marine environments, including marine algae [1,3], seawater [4,7], marine sediments [8,11], sponges [12,14] and marine invertebrates [15]. Members of *Flagellimonas* are characterized as Gram-stain-negative, non-spore-forming, rod-shaped and NaCl-requiring bacteria that consistently exhibit catalase and oxidase activities. They are generally strictly aerobic, although facultative aerobic growth has been rarely reported. Despite the genus name implying motility, most species are non-motile and lack flagella [1,3,15]. Chemotaxonomic features include menaquinone-6 (MK-6) as the predominant respiratory quinone, iso-C_15:0_, iso-C_15:1_ G and iso-C_17:0_ 3-OH as major fatty acids and phosphatidylethanolamine (PE) as the major polar lipid [1,3,15]. As part of our ongoing taxonomic investigation of macroalgae-associated microbiota [16,20], we isolated and characterized two novel *Flagellimonas*-affiliated strains from the phycosphere of marine algae. Their taxonomic status was elucidated using a polyphasic approach incorporating phenotypic, genotypic and phylogenetic analyses.

## Strain isolation

Strains S174ᵀ and W118ᵀ were isolated from the marine green alga *Ulva prolifera* and the marine red alga *Schizymenia dubyi*, respectively, collected from Namhae-gun (34° 44′ 28.9″ N 128° 02′ 53.7″ E) and Taean-gun (36° 53′ 30.6″ N 126° 11′ 52.8″ E) in South Korea, as previously described with minor modifications [20]. The algae were thoroughly washed with artificial seawater (ASW; 20.0 g NaCl, 2.9 g MgSO_4_, 4.5 g MgCl_2_·6H_2_O, 0.6 g KCl, 1.8 g CaCl_2_·2H_2_O per litre) by vigorous vortexing to remove surface-associated micro-organisms. The washed samples were homogenized using a T10 basic homogenizer (IKA, Germany) and serially diluted in ASW. Aliquots (100 µl) of each dilution were spread onto marine agar (MA; MBcell, South Korea) and incubated aerobically at 25 °C for 3 days. Colonies grown on MA were used as templates for PCR amplification with universal primers 27F (5′-AGA GTT TGA TCM TGG CTC AG-3′) and 1492R (5′-TAC GGY TAC CTT GTT ACG ACT T-3′) [20]. The PCR products were digested with restriction enzymes HaeIII and HhaI, and the resulting fragments were separated on a 2% (w/v) agarose gel. Amplicons with distinctive patterns were partially sequenced using primer 340F (5′-CCT ACG GGA GGC AGC AG-3′) [20] by Macrogen (South Korea), and the resulting 16S rRNA gene sequences were compared to those of type strains using the nucleotide similarity search tool in EzBioCloud (http://www.ezbiocloud.net/identify) [21]. Two strains, S174ᵀ and W118ᵀ, showing low similarity to known species of *Flagellimonas*, were selected for further taxonomic analysis. These strains were routinely cultured in marine broth (MB; MBcell) at 25 °C for 3 days and preserved at −80 °C in MB supplemented with 15% (v/v) glycerol.

## Phylogeny based on 16S rRNA gene sequences

PCR amplicons of strains S174ᵀ and W118ᵀ, initially generated using primers F1 and R13, were further sequenced with the universal 16S rRNA primers 518R (5′-ATT ACC GCG GCT GCT GG-3′) and 805F (5′-GAT TAG ATA CCC TGG TAG TC-3′) [20]. Nearly complete 16S rRNA gene sequences were obtained for strains S174ᵀ (1,449 bp) and W118ᵀ (1,466 bp) by assembling reads obtained with primers 340F, 518R and 805F. Sequence similarities between the two strains and their phylogenetically related type strains were calculated using the nucleotide similarity search tool on the EzBioCloud server. The 16S rRNA gene sequences of strains S174ᵀ and W118ᵀ, along with those of related type strains, were aligned using the secondary-structure-aware Infernal aligner (version 1.1.4) [22], and phylogenetic trees were reconstructed using neighbour-joining (NJ), maximum-parsimony (MP) and maximum-likelihood (ML) methods implemented in MEGA11 [23]. The NJ tree was constructed using the Kimura two-parameter model with uniform rates, the ML tree with the nearest-neighbour-interchange heuristic method and the MP tree with the subtree-pruning-regrafting search method; all analyses were performed with complete gap deletion. Robustness of the phylogenies was assessed by bootstrap analysis with 1,000 replicates.

**Fig. 1.**
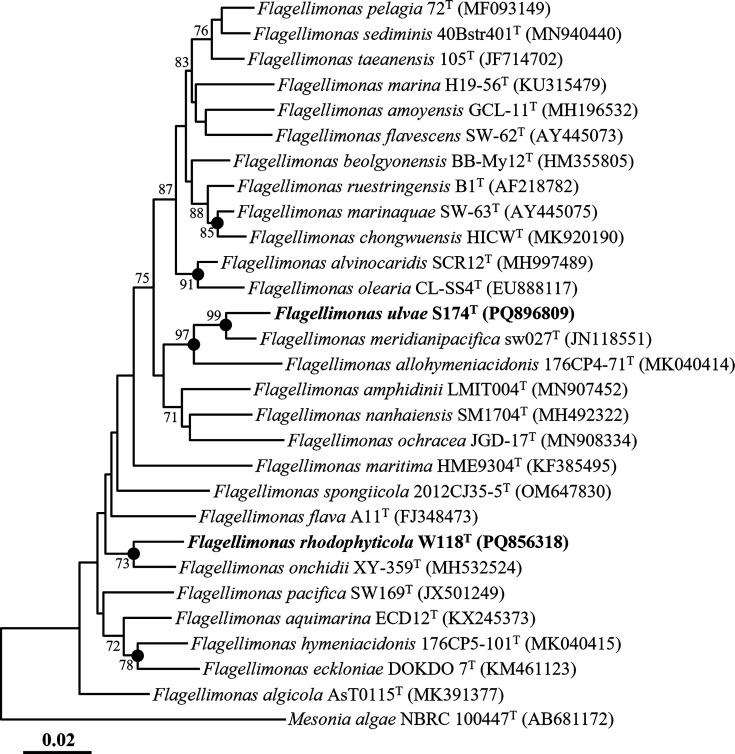
NJ tree showing the phylogenetic relationships of strains S174ᵀ and W118ᵀ with closely related type strains based on 16S rRNA gene sequences. Bootstrap values ≥70% from 1,000 replicates are shown at the corresponding nodes. Filled circles (●) indicate nodes also supported by both ML and MP methods. *Mesonia algae* NBRC 100447ᵀ (AB681172) was used as the outgroup. Scale bar represents 0.02 substitutions per nucleotide position.

The 16S rRNA gene sequence similarity between strains S174ᵀ and W118ᵀ was 93.6%, well below the commonly accepted species delineation threshold of 98.5–98.7% [24], indicating that the two strains represent distinct species. In pairwise comparisons, strain S174ᵀ showed the highest similarity to *Flagellimonas meridianipacifica* SW027ᵀ (97.2%) and *Flagellimonas allohymeniacidonis* 176CP4-71ᵀ (96.8%), while strain W118ᵀ was most similar to *Flagellimonas onchidii* XY-359ᵀ (97.9%), *Flagellimonas spongiicola* 2012CJ35-5ᵀ (96.6%) and *Flagellimonas hymeniacidonis* 176CP5-101ᵀ (96.5%), suggesting their classification as novel species distinct from previously reported species. Phylogenetic analysis based on 16S rRNA gene sequences using the NJ algorithm placed strains S174ᵀ and W118ᵀ in distinct lineages within the genus *Flagellimonas* (Fig. 1), which was corroborated by ML and MP trees (Fig. S1, available in the online Supplementary Material). Together, these comparative and phylogenetic results support the proposal that strains S174ᵀ and W118ᵀ represent two novel species within the genus *Flagellimonas*.

## Whole-genome sequencing and genome-based phylogeny

Genomic DNA of strains S174ᵀ and W118ᵀ was extracted from cultures grown in MB using the Wizard Genomic DNA Purification Kit (Promega, USA). Whole-genome sequencing was performed using the Oxford Nanopore MinION platform (ONT, UK), and *de novo* assembly was conducted with Flye (version 3.0.0) [25]. Genome quality was assessed with CheckM2 (version 1.0.2) [26]. The assemblies yielded complete single circular chromosomes of 4,127 kb for strain S174ᵀ and 3,679 kb for strain W118ᵀ, with average coverages of ~72× and 250×, respectively. Both genomes contained two copies of the 16S rRNA gene. In strain S174ᵀ, one copy was identical to the PCR-based sequence, while the other showed 99.9% identity. In strain W118ᵀ, both copies were identical to the PCR-based sequence. These results confirm that the genome sequencing and assembly of both strains were accurate and free from contamination. Genome quality assessments indicated completeness values of 100% for strain S174ᵀ and 99.6% for strain W118ᵀ, with contamination rates of 0.6 and 0%, respectively, satisfying the criteria for high-quality genomes (≥90% completeness and ≤10% contamination) [26].

Phylogenomic analysis of strains S174ᵀ and W118ᵀ and their closest type strains was performed using the Genome Taxonomy Database Toolkit (GTDB-Tk), based on the concatenated amino acid sequences of 120 ubiquitous single-copy marker genes (bac120 marker set) [27]. The aligned sequences were used to construct an ML phylogenomic tree in MEGA11 with bootstrap support calculated from 1,000 replicates. Genome relatedness was assessed by calculating average nucleotide identity (ANI) using the Orthologous ANI Tool (version 0.93.1; www.ezbiocloud.net/tools/orthoani) [28] and digital DNA–DNA hybridization (dDDH) values using the genome-to-genome distance calculator (GGDC 3.0; https://ggdc.dsmz.de/ggdc.php) with formula 2 [29].

**Fig. 2.**
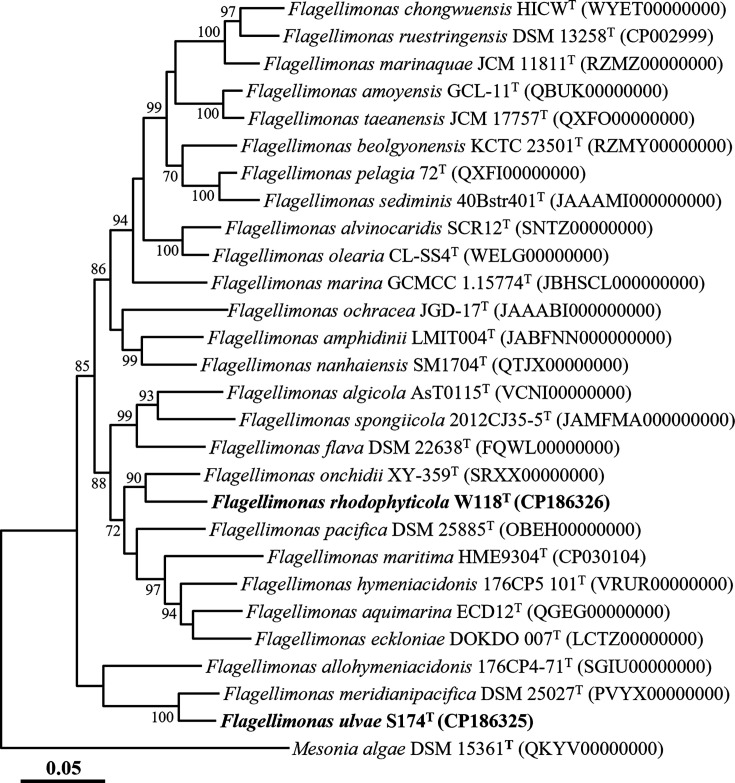
ML tree showing the phylogenetic relationships of strains S174ᵀ and W118ᵀ with closely related type strains, based on concatenated amino acid sequences of 120 bacterial marker genes (bac120 marker set) generated using GTDB-Tk. Bootstrap values ≥70% from 1,000 replicates are shown at the corresponding nodes. *Mesonia algae* DSM 15361ᵀ (QKYV00000000) was used as the outgroup. Scale bar indicates 0.05 substitutions per amino acid position.

The genome-based phylogenomic tree showed that strains S174ᵀ and W118ᵀ formed distinct clades with *F. meridianipacifica* DSM 25027ᵀ and *F. onchidii* XY-359ᵀ, respectively (Fig. 2), confirming their affiliation with the genus *Flagellimonas*, as also supported by 16S rRNA gene-based phylogenies. The ANI and dDDH values between strains S174ᵀ and W118ᵀ were 72.7 and 17.5%, respectively, falling well below the accepted species delineation thresholds (~95% ANI and ~70% dDDH) [24], indicating that they represent distinct species. Furthermore, the ANI and dDDH values between each strain and all described species were below 84.6 and 28.2%, respectively (Table S1), further supporting their status as novel species. Based on phylogenetic and genomic analyses, *F. meridianipacifica* JCM 17861ᵀ and *F. onchidii* KCTC 72218ᵀ were selected as reference strains for comparative analyses of genome features, phenotypic characteristics and fatty acid profiles.

## Genomic features and algal symbiosis-associated genes

The whole-genome sequences of strains S174ᵀ and W118ᵀ were submitted to GenBank and annotated using the NCBI Prokaryotic Genome Annotation Pipeline. The genomes of S174ᵀ and W118ᵀ were predicted to contain 3,865 and 3,314 genes, respectively, including 3,783 and 3,260 protein-coding sequences, 2 complete rRNA operons (16S, 23S, 5S) and 51 and 40 tRNA genes representing all 20 amino acids (Table 1). These genomic features, including genome size, total gene count, protein-coding genes and tRNA genes, are comparable to those of closely related *Flagellimonas* species.

**Table 1.** General genomic features of strains S174^T^ and W118^T^ and closely related type strains of the genus *Flagellimonas* Taxa: 1, strain S174^T^ (CP186325); 2, strain W118^T^ (CP186326); 3, *F. meridianipacifica* DSM 25027^T^ (PVYX00000000); 4, *F. onchidii* XY-359^T^ (SRXX00000000). The genomes of strains S174^T^ and W118^T^ were sequenced in this study.

Feature	1	2	3	4
Genome status* (no. of contigs)	C (1)	C (1)	D (4)	D (57)
Total genome size (kb)	4,127	3,679	4,376	4,208
G+C content (mol%)	39.1	39.1	39.7	39.1
No. of total genes	3,865	3,314	4,076	3,863
No. of protein-coding sequences	3,783	3,260	4,024	3,788
No. of non-coding RNA genes	4	4	4	4
No. of rRNA (16S, 23S, 5S) genes	2, 2, 2	2, 2, 2	1, 1, 1	1, 1, 3
No. of tRNA genes	51	40	37	36
No. of total CAZy† genes	75	85	82	94
Glycoside hydrolases (GHs)	38	49	44	52
Glycosyltransferases (GTs)	30	25	31	35
Carbohydrate esterases (CEs)	0	3	2	0
Polysaccharide lyases (PLs)	5	4	1	2
Carbohydrate-binding modules (CBMs)	2	4	4	5
Auxiliary activities (AAs)	0	0	0	0

*C, complete; D, draft.

Algae are rich in polysaccharides, which serve as key structural and functional components of their extracellular matrices, cell walls and storage compounds. Accordingly, the ability to degrade diverse algal polysaccharides is considered an important trait of heterotrophic bacteria associated with marine algae. To predict the polysaccharide-degrading potential of strains S174ᵀ and W118ᵀ, as well as closely related *Flagellimonas* strains, their predicted protein sequences were analysed using the dbCAN3 meta server (https://bcb.unl.edu/dbCAN2/blast.php) [30] for the identification of carbohydrate-active enzymes (CAZys). CAZys are categorized into six major classes: glycoside hydrolases, glycosyltransferases, carbohydrate esterases, polysaccharide lyases, carbohydrate-binding modules and auxiliary activities. The genomes of strains S174ᵀ and W118ᵀ were predicted to encode 75 and 85 CAZy-related genes, respectively – comparable to those found in other *Flagellimonas* species not isolated from algal phycospheres – suggesting that the ability to utilize algal polysaccharides may be an intrinsic metabolic feature of this genus.

**Fig. 3.**
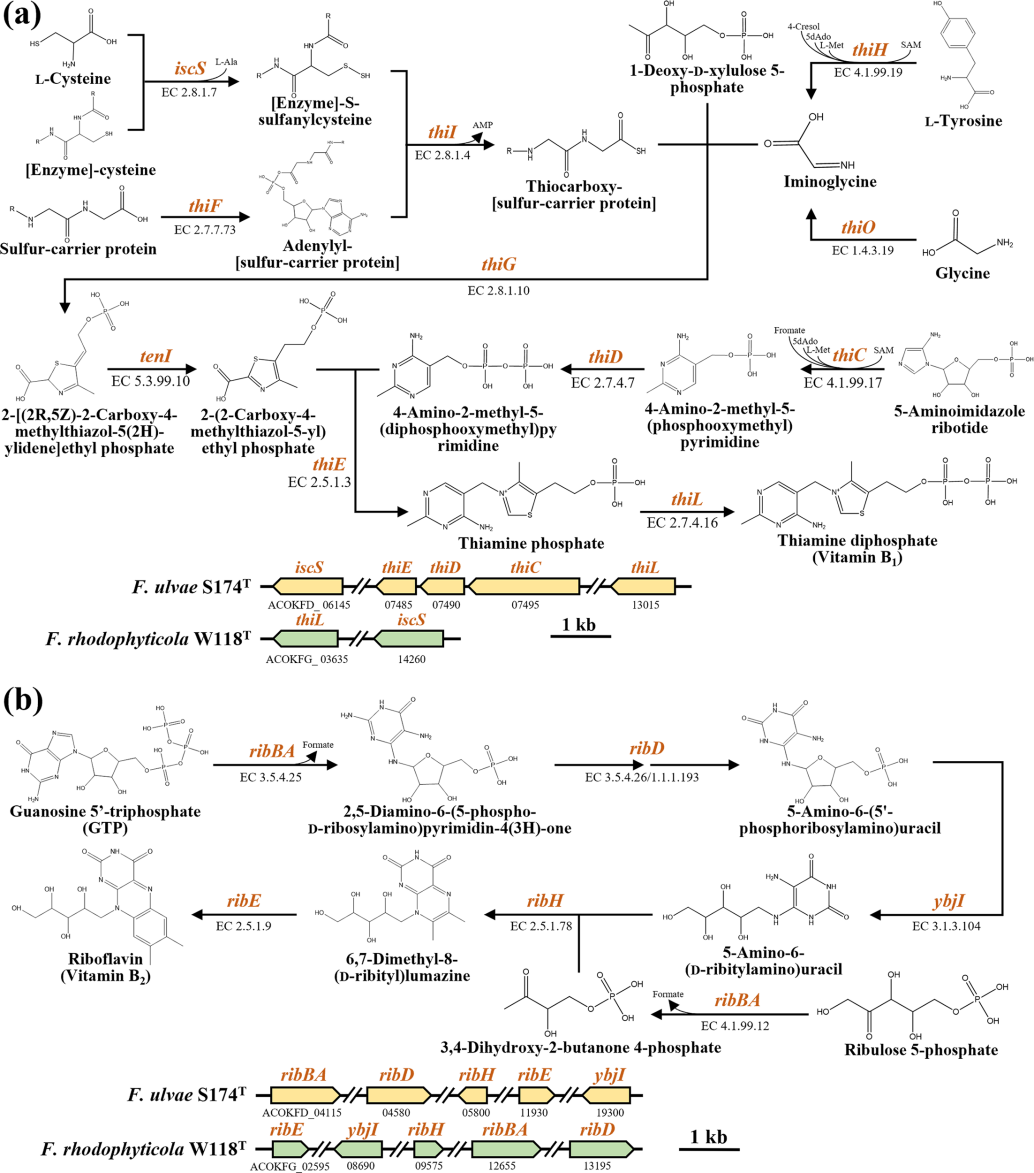
Biosynthetic pathways and corresponding genes of (a) thiamine diphosphate (vitamin B_1_) and (b) riboflavin (vitamin B_2_) identified in strains S174^T^ and W118^T^. Genes: *iscS*, cysteine desulfurase; *thiF*, sulphur carrier protein ThiS adenylyltransferase; *thiI*, tRNA uracil 4-sulfurtransferase; *thiH*, 2-iminoacetate synthase; *thiO*, glycine oxidase; *thiG*, thiazole synthase; *tenI*, thiazole tautomerase; *thiD*, phosphooxymethylpyrimidine kinase; *thiC*, phosphomethylpyrimidine synthase; *thiE*, thiamine phosphate synthase; *thiL*, thiamine-monophosphate kinase; *ribBA*, GTP cyclohydrolase II/3,4-dihydroxy 2-butanone 4-phosphate synthase; *ribD*, diaminohydroxyphosphoribosylaminopyrimidine deaminase/5-amino-6-(5-phosphoribosylamino)uracil reductase; *ybjI*, 5-amino-6-(5-phospho-d-ribitylamino)uracil phosphatase; *ribH*, 6,7-dimethyl-8-ribityllumazine synthase; *ribE*, riboflavin synthase.

Marine bacteria residing in the phycosphere can affect their algal hosts by producing various metabolites, including vitamins, siderophores, compatible solutes and essential nutrients [31,32]. To explore the symbiotic potential of strains S174ᵀ and W118ᵀ, genome analyses were conducted using blastp searches with reference protein sequences from the UniProt database (https://www.uniprot.org). Strain S174ᵀ was found to possess *iscS* and *thiCDEL*, genes involved in thiamine diphosphate (vitamin B_1_) biosynthesis from precursors including l-cysteine, l-tyrosine, glycine, 1-deoxy-d-xylulose 5-phosphate and 5-aminoimidazole ribotide, but lacked *thiEFGHIO*, while strain W118ᵀ harbours only *iscS* and *thiL* (Fig. 3a), indicating that both strains are unlikely to synthesize vitamin B_1_ independently and may depend on metabolic interactions with other organisms. By contrast, both strains contained a complete set of riboflavin (vitamin B_2_) biosynthetic genes (*ribABDEH* and *ybiI*) (Fig. 3b), enabling the synthesis of vitamin B_2_ from guanosine 5′-triphosphate and ribulose-5-phosphate. These genomic traits suggest that strains S174ᵀ and W118ᵀ may contribute to algal growth in the phycosphere by supplying essential B vitamins.

## Morphology and phenotypic characteristics

The growth of strains S174ᵀ and W118ᵀ was tested on various agar media (MBcell), including MA, nutrient agar (NA), Luria–Bertani (LB) agar, tryptic soy agar (TSA) and Reasoner’s 2A (R2A) agar, each containing ~2% (w/v) NaCl. Plates were incubated at 25 °C for 3 days. Temperature-dependent growth was assessed on MA at 5–50 °C in 5 °C intervals, while pH tolerance was evaluated in MB adjusted to pH 5.0–11.0 in 1-unit increments at 25 °C for 3 days. Buffer systems included sodium citrate (pH 5.0), sodium phosphate (pH 6.0–8.0) and sodium carbonate/bicarbonate (pH 9.0–11.0), with pH adjusted post-autoclaving if needed. Salt tolerance was determined in MB supplemented with NaCl concentrations ranging from 0 to 10% (w/v) in 1% increments. Anaerobic growth was tested on MA at 25 °C for 21 days using the GasPak Plus system (BBL, USA).

Cell morphology and motility of strains S174ᵀ and W118ᵀ were examined by phase-contrast microscopy (Zeiss Axio Scope.A1; Carl Zeiss, Germany) after cultivation on MA at 25 °C for 2 days. For transmission electron microscopy (JEM-F200; JEOL, Japan), cells were placed on Formvar-coated copper grids (Electron Microscopy Science, USA), stained with UranyLess (Electron Microscopy Science, USA) for 1 min [33] and visualized. Gram staining was performed using a commercial kit (bioMérieux, France) according to the manufacturer’s instructions. Catalase activity was determined by bubble formation in 3% (v/v) hydrogen peroxide (Junsei, Japan), and oxidase activity by colour change upon oxidation of 1% (w/v) tetramethyl-*p*-phenylenediamine (Merck, USA). Phenotypic traits of strains S174ᵀ and W118ᵀ, along with reference strains *F. meridianipacifica* JCM 17861ᵀ and *F. onchidii* KCTC 72218ᵀ, were assessed at their optimal growth temperatures. Hydrolysis of casein (1% skim milk), starch (1%), aesculin (0.1%), l-tyrosine (0.5%), Tween 20 (1%) and Tween 80 (1%) was tested on MA using standard procedures [34]. Additional biochemical and enzymatic characteristics were determined using API 20NE kits following the manufacturer’s protocol.

Strains S174ᵀ and W118ᵀ showed robust growth on MA but failed to grow on NA, TSA, LB agar or R2A agar. Both strains were Gram-stain-negative, non-flagellated rods, with cell dimensions of 0.4–0.5×2.9–3.0 µm for strain S174ᵀ and 0.4–0.5×1.6–2.1 µm for strain W118ᵀ. Outer membrane vesicle-like structures were observed on the surface of strain S174ᵀ (Fig. S2). Neither strain grew under anaerobic conditions, indicating an obligately aerobic lifestyle. Strains S174ᵀ and W118ᵀ shared many phenotypic, physiological and biochemical characteristics with closely related *Flagellimonas* species, including rod-shaped morphology, motility, nitrate reduction, activity of oxidase, catalase, urease and *β*-galactosidase and indole production. They also showed similar profiles for glucose fermentation, tyrosine, Tween 20 and gelatin hydrolysis and assimilation of l-arabinose, d-mannitol, capric acid, malic acid, trisodium citrate and phenylacetic acid. However, some differences were observed in growth ranges, arginine dehydrolase activity, substrate assimilation (e.g. d-glucose, d-mannose, *N*-acetylglucosamine, d-maltose, potassium gluconate and adipic acid) and hydrolytic activities (e.g. starch, Tween 80, aesculin and casein), which distinguish them from related *Flagellimonas* species (Table 2).

**Table 2.** Differential characteristics between strains S174^T^ and W118^T^ and closely related type strains of the genus *Flagellimonas* Taxa: 1, strain S174^T^ (this study); 2, strain W118^T^ (this study); 3, *F. meridianipacifica* JCM 17861^T^ [4]; 4, *F. onchidii* KCTC 72218^T^ [15]. All strains are positive for the following characteristics: activity* of oxidase, catalase and *β*-galactosidase and tyrosine hydrolysis*. All strains are negative for the following characteristics: flagella motility, urease activity*, nitrate reduction*, indole production*, glucose fermentation*, hydrolysis* of Tween 20 and gelatin and assimilation* of l-arabinose, d-mannitol, capric acid, malic acid, trisodium citrate and phenylacetic acid. Symbols: +, positive; −, negative; *w,* weakly positive.

Characteristic	1	2	3	4
Isolation source	Marine alga	Marine alga	Seawater	Marine invertebrate
Colony colour	Light orange	Greyish yellow	Orange	Cream
Growth range (optimum) of:				
Temperature (°C)	20–30 (25)	20–30 (25)	10–42 (37)	15–37 (30)
pH	7.0–10.0 (8.0)	8.0–9.0 (8.0)	6.0–9.0 (7.0)	6.0–8.5 (7.5)
NaCl (w/v, %)	1.0–4.0 (2.0)	1.0–4.0 (2.0)	1.0–9.0 (3.0)	0.5–4.5 (2.5)
Hydrolysis* of:				
Starch, casein	+	−	+	−
Tween 80	+	*w*	+	+
Aesculin	+	+	*+*	−
Arginine dehydrolase activity	−	−	−	+
Assimilation* of:				
d-Glucose, d-maltose	−	−	−	*+*
d-Mannose	−	+	−	+
*N*-Acetylglucosamine	−	*w*	−	*w*
Potassium gluconate	−	+	−	−
Adipic acid	*w*	−	−	−
Major fatty acids (>10%)*	iso-C_15:1_ G, iso-C_15:0_, iso-C_17:0_ 3-OH	iso-C_15:1_ G, iso-C_15:0_, iso-C_17:0_ 3-OH	iso-C_15:1_ G, iso-C_15:0_, iso-C_17:0_ 3-OH	iso-C_15:1_ G, iso-C_15:0_, iso-C_17:0_ 3-OH
Polar lipids†	PE, AL, L	PE, AL, L	PE, AL, PL, L	PE, L

*These analyses were conducted under the same conditions in this study.

†PE, phosphatidylethanolamine; AL, unidentified aminolipid; PL, unidentified phospholipid; L, unidentified lipid.

## Chemotaxonomic characteristics

Respiratory isoprenoid quinones of strains S174ᵀ and W118ᵀ were extracted from cultures grown in MB at 25 °C for 3 days. Quinone profiles were analysed using HPLC (LC-20A, Shimadzu, Japan) equipped with a reversed-phase column (250×4.6 mm, Kromasil, Akzo Nobel, Netherlands) and a diode-array detector (SPD-M20A, Shimadzu), employing a methanol-isopropanol (2:1, v/v) solvent system at a flow rate of 1 ml min^−1^, as previously described [35]. For cellular fatty acid analysis, strains S174ᵀ, W118ᵀ and two reference strains were cultured in MB at their optimal temperatures. Cells were harvested during the exponential phase (OD_600_=0.7–0.8), and fatty acid methyl esters were prepared using the standard MIDI protocol (Sherlock Microbial Identification System, version 6.2B), including saponification, methylation and extraction. Fatty acids were analysed by GC (Hewlett Packard 6890, USA) and identified using the RTSBA6 database (Sherlock version 6.0B) [36]. Polar lipids were examined by two-dimensional TLC from exponentially grown cells, following the method of Minnikin *et al.* [35]. Detection reagents included ethanolic molybdophosphoric acid (total polar lipids), ninhydrin (aminolipids), Dittmer-Lester reagent (phospholipids) and *α*-naphthol/sulphuric acid (glycolipids). PE was identified by comparison with authentic standards (Sigma-Aldrich, USA).

Strains S174ᵀ and W118ᵀ contained MK-6 as the sole respiratory quinone, consistent with other members of the genus *Flagellimonas* [1,3,15]. The major fatty acids (>10%) in both strains and their closely related type strains were iso-C_15:1_ G, iso-C_15:0_ and iso-C_17:0_ 3-OH (Table S2). Although the overall fatty acid profiles of strains S174ᵀ and W118ᵀ were similar to those of related type strains, some variation was observed in the relative abundances of individual components. Both strains also contained PE as a major polar lipid (Fig. S3), which is a characteristic feature of the genus *Flagellimonas* [1,3,15]. Additionally, two unidentified aminolipids and three unidentified lipids were detected in both strains.

## Taxonomic conclusion

Collectively, the phylogenetic, phenotypic, physiological, chemotaxonomic and genomic evidence supports the classification of strains S174ᵀ and W118ᵀ as two distinct novel species within the genus *Flagellimonas*. Accordingly, the names *Flagellimonas ulvae* sp. nov. and *Flagellimonas rhodophyticola* sp. nov. are proposed for strains S174ᵀ and W118ᵀ, respectively.

## Description of *Flagellimonas ulvae* sp. nov.

*Flagellimonas ulvae* (ul′vae. L. gen. n. *ulvae*, of a seaweed).

Colonies on MA are light orange, circular, convex and smooth. Cells are Gram-stain-negative, non-spore-forming, strictly aerobic and non-motile rods. Growth occurs at 20–30 °C (optimum, 25 °C), pH 7.0–10.0 (optimum, pH 8.0) and 1.0–4.0% (w/v) NaCl (optimum, 2.0%). Oxidase- and catalase-positive. Nitrate reduction, indole production and glucose fermentation are not observed. Aesculin, tyrosine, starch, Tween 80 and casein are hydrolysed, whereas Tween 20 and gelatin are not hydrolysed. *β*-Galactosidase activity is present, while urease and arginine dihydrolase activities are absent. Assimilates adipic acid (weakly), but not d-glucose, d-mannose, *N*-acetylglucosamine, d-maltose, potassium gluconate, l-arabinose, d-mannitol, capric acid, malic acid, trisodium citrate and phenylacetic acid. The major respiratory quinone is MK-6. Major fatty acids (>10%) include iso-C_15:1_ G, iso-C_15:0_ and iso-C_17:0_ 3-OH. The major polar lipid is PE.

The type strain is S174^T^ (=KACC 24029^T^=JCM 37803^T^), isolated from a marine green alga *U. prolifera*, collected in South Korea. The genome size and DNA G+C content are 4,127 kb and 39.1 mol% (calculated from the whole-genome sequence), respectively. The GenBank accession numbers of the 16S rRNA gene and genome sequences of strain S174^T^ are PQ896809 and CP186325, respectively.

## Description of *Flagellimonas rhodophyticola* sp. nov.

*Flagellimonas rhodophyticola* [rho.do.phy.ti′co.la. N.L. neut. pl. n. *Rhodophyta*, the division of the red algae; L. suffix. -*cola* (from L. masc. or fem. n. *incola*), inhabitant, dweller; N.L. fem. n. *rhodophyticola*, inhabitant of *Rhodophyta*].

Colonies on MA are greyish yellow, circular, convex and smooth. Cells are Gram-stain-negative, non-spore-forming, strictly aerobic and non-motile rods. Growth occurs at 20–30 °C (optimum, 25 °C), pH 8.0–9.0 (optimum, pH 8.0) and 1.0–4.0% (w/v) NaCl (optimum, 2.0%). Oxidase- and catalase-positive. Nitrate reduction, indole production and glucose fermentation are negative. Aesculin, tyrosine and (weakly) Tween 80 are hydrolysed, whereas Tween 20, gelatin, casein and starch are not. *β*-Galactosidase activity is present, while urease and arginine dihydrolase activities are absent. Assimilates d-mannose, *N*-acetylglucosamine (weakly) and potassium gluconate, but not d-glucose, d-maltose, adipic acid, l-arabinose, d-mannitol, capric acid, malic acid, trisodium citrate and phenylacetic acid. The major respiratory quinone is MK-6. Major fatty acids (>10%) include iso-C_15:1_ G, iso-C_15:0_ and iso-C_17:0_ 3-OH. The major polar lipid is PE.

The type strain is W118^T^ (=KACC 24030^T^=JCM 37802^T^), isolated from a marine red alga *S. dubyi*, collected in South Korea. The genome size and DNA G+C content are 3,679 kb and 39.1 mol% (calculated from the whole-genome sequence), respectively. The GenBank accession numbers of the 16S rRNA gene and genome sequences of strain W118^T^ are PQ856318 and CP186326, respectively.

## Supplementary material

10.1099/ijsem.0.006996 Uncited Supplementary Material 1.

## Abbreviations

ANI: average nucleotide identity
ASW: artificial seawater
CAZy: carbohydrate-active enzyme
dDDH: digital DNA–DNA hybridization
GTDB-Tk: Genome Taxonomy Database Toolkit
LB: Luria–Bertani
MA: marine agar
MB: marine broth
MK-6: menaquinone-6
ML: maximum-likelihood
MP: maximum-parsimony
NA: nutrient agar
NJ: neighbour-joining
PE: phosphatidylethanolamine
R2A: Reasoner’s 2A
TSA: tryptic soy agar
