# Supplementary material for: Flagellimonas ulvae sp. nov. and Flagellimonas rhodophyticola sp. nov., isolated from marine algae
Source: Int J Syst Evol Microbiol. 2025 Dec 9;75(12):006996. doi: 10.1099/ijsem.0.006996 (PMC12688033; doi:10.1099/ijsem.0.006996)
Supplement: Uncited Supplementary Material 1. [file ijsem-75-06996-s001.pdf]

Supplementary Information

Fig. S1. Maximum-likelihood (ML, a) and maximum-parsimony (MP, b) trees showing the phylogenetic relationships of strains S174^{T} and W118^{T} with closely related taxa based on 16S rRNA gene sequences. Bootstrap values ≥70% (from 1,000 replicates) are shown at the nodes. *Mesonia algae* NBRC 100447^{T} (AB681172) was used as the outgroup. Scale bars in ML and MP trees indicate nucleotide substitutions per site and total substitutions across the full sequence, respectively.

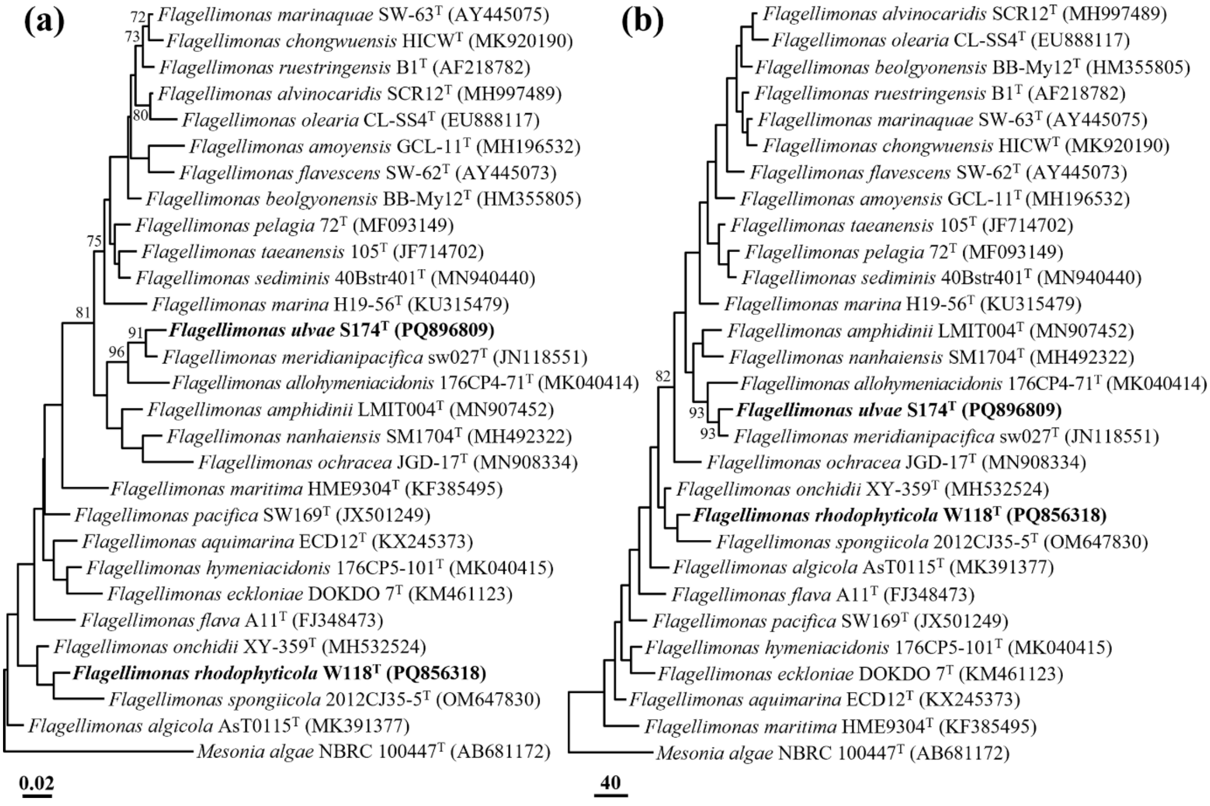

Fig. S2. Transmission electron micrographs of strains S174^T (a) and W118^T (b), cultured on marine agar at 25 °C for 2 days and negatively stained with UranyLess. Arrows in panel A indicate putative outer membrane vesicles on the surface of strain S174^T.

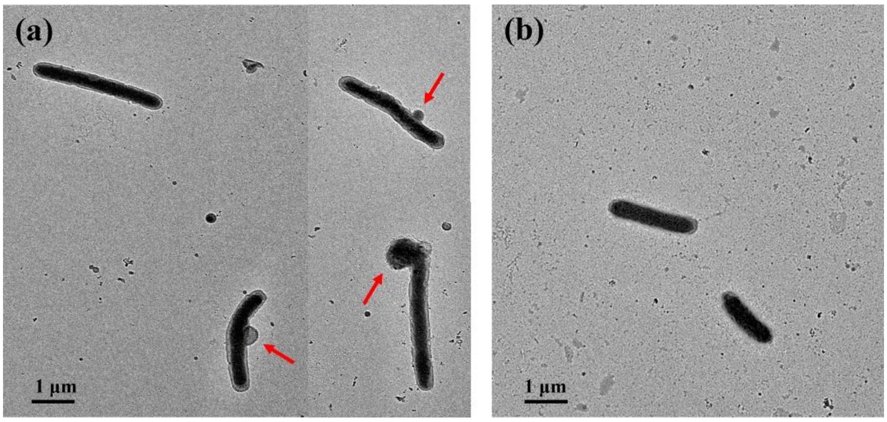

Fig. S3. Two-dimensional thin-layer chromatograms (TLC) showing the polar lipid profiles of strains S174^T and W118^T. Solvent systems: (I) chloroform-methanol-water (65:25:4, v/v/v) and (II) chloroform-acetic acid-methanol-water (80:15:12:4, v/v/v/v). The TLC plates were sprayed with 10% ethanolic molybdophosphoric acid (a), ninhydrin (b), Dittmer-Lester (c), and α-naphthol/sulfuric acid (d) reagents for the detection of total polar lipids, aminolipids, phospholipids, and glycolipids, respectively. PE, phosphatidylethanolamine; AL, unidentified aminolipid; L, unidentified polar lipid.

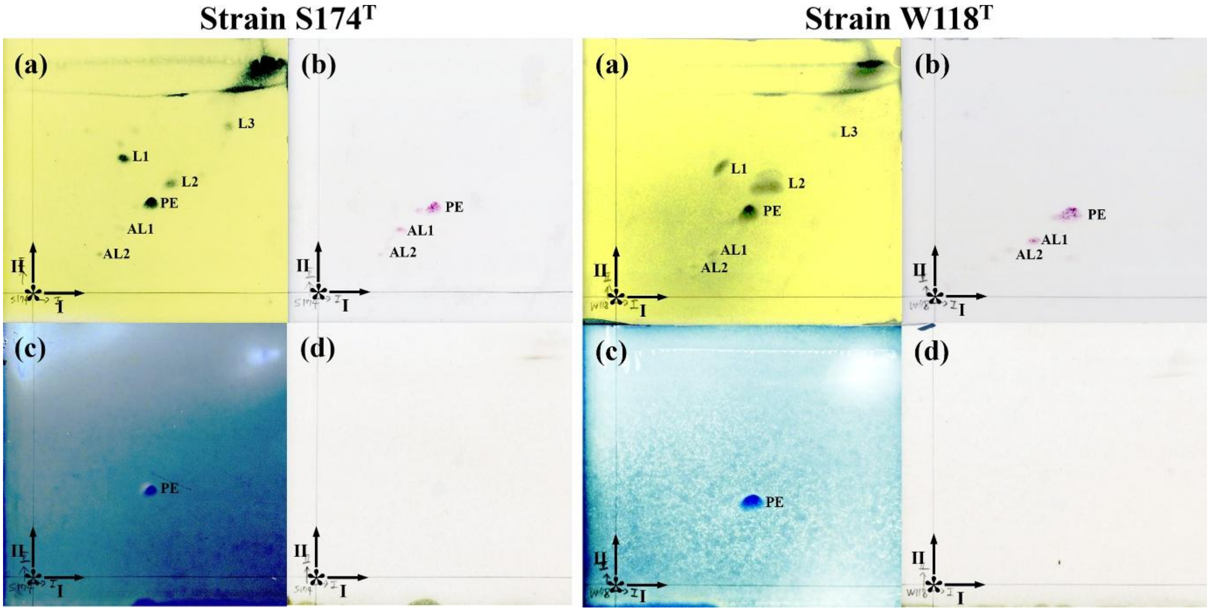

Table S1. Genome relatedness among strains S174^T and W118^T and their closely related type strains of the genus *Flagellimonas*

Taxa: 1, strain S174^T (CP186325); 2, strain W118^T (CP186326); 3, *F. meridianipacifica* DSM 25027^T (PVYX00000000); 4, *F. onchidii* XY-359^T (SRXX00000000); 5, *F. allohymeniacidonis* 176CP4-71^T (SGIU00000000); 6, *F. pacifica* DSM 25885^T (OBEH00000000); 7, *F. maritima* HME9304^T (CP030104); 8, *F. flava* DSM 22638^T (FQWL00000000).

		dDDH† value (%)							
		**1**	**2**	**3**	**4**	**5**	**6**	**7**	**8**
ANI† value (%)	**1**	–	17.5	28.2	17.4	17.4	17.6	17.4	17.5
	**2**	72.7	–	17.3	20.2	17.3	20.1	18.3	18.1
	**3**	84.6	72.6	–	17.5	17.4	17.8	17.2	17.2
	**4**	72.7	77.5	72.7	–	17.5	20.1	18.0	18.0
	**5**	73.3	72.8	73.4	73.0	–	17.4	17.4	17.1
	**6**	73.0	77.1	73.0	76.9	73.2	–	18.9	18.3
	**7**	72.7	73.9	72.4	73.8	72.6	74.7	–	17.7
	**8**	72.4	73.9	72.3	73.9	72.5	72.3	73.1	–

†ANI, average nucleotide identity; dDDH, digital DNA-DNA hybridization.

Table S2. Cellular fatty acid compositions (%) of strains S174^T and W118^T and closely related type strains of the genus *Flagellimonas*

Taxa: 1, strain S174^T; 2, strain W118^T; 3, *F. meridianipacifica* JCM 17861^T; 4, *F. onchidii* KCTC 72218^T. All data were obtained from this study. Data are expressed as percentages of the total fatty acids, and fatty acids constituting less than 1.0% in all strains are not indicated. Major components (>10.0%) are highlighted in bold; symbols: tr, trace amount (<1.0%); –, not detected.

Fatty acid	1	2	3	4
Saturated:				
$C_{10:0}$	1.3	1.1	–	–
$C_{12:0}$	1.7	1.4	tr	tr
$C_{14:0}$	1.2	tr	1.3	tr
$C_{16:0}$	1.4	1.5	5.3	1.1
$C_{18:0}$	tr	tr	2.8	–
Unsaturated:				
$C_{18:1}$ $\omega 9c$	–	tr	3.3	–
Branched:				
iso-$C_{10:0}$	tr	tr	1.6	tr
iso-$C_{13:0}$	tr	tr	tr	1.2
iso-$C_{15:1}$ G	**32.5**	**34.7**	**25.6**	**28.1**
iso-$C_{15:0}$	**19.1**	**20.0**	**22.8**	**28.7**
iso-$C_{16:0}$	tr	tr	1.11	tr
anteiso-$C_{15:0}$	1.3	1.4	tr	tr
Hydroxy:				
iso-$C_{12:0}$ 3-OH	1.3	1.1	tr	1.3
iso-$C_{15:0}$ 3-OH	4.5	4.4	5.2	6.1
iso-$C_{16:0}$ 3-OH	2.7	2.5	1.2	–
$C_{16:0}$ 3-OH	1.1	tr	1.6	–
iso-$C_{17:0}$ 3-OH	**14.4**	**13.9**	**15.9**	**15.9**
Summed feature*:				
3 ($C_{16:1}$ $\omega 6c$ and/or $C_{16:1}$ $\omega 7c$)	6.1	5.5	6.5	9.4

*Summed features are fatty acids that cannot be resolved reliably from another fatty acid using the chromatographic conditions chosen. The MIDI system groups these fatty acids together as one feature with a single percentage of the total.
